# Order in Spontaneous Behavior

**DOI:** 10.1371/journal.pone.0000443

**Published:** 2007-05-16

**Authors:** Alexander Maye, Chih-hao Hsieh, George Sugihara, Björn Brembs

**Affiliations:** 1 Universitätsklinikum Hamburg-Eppendorf, Zentrum für Experimentelle Medizin, Institut für Neurophysiologie und Pathophysiologie, Hamburg, Germany; 2 Scripps Institution of Oceanography, University of California San Diego, La Jolla, California, United States of America; 3 Freie Universität Berlin, Institut für Biologie–Neurobiologie, Berlin, Germany; Centre de Recherches su la Cognition Animale-Centre National de la Recherche Scientifique and Université Paul Sabatier, France

## Abstract

Brains are usually described as input/output systems: they transform sensory input into motor output. However, the motor output of brains (behavior) is notoriously variable, even under identical sensory conditions. The question of whether this behavioral variability merely reflects residual deviations due to extrinsic random noise in such otherwise deterministic systems or an intrinsic, adaptive indeterminacy trait is central for the basic understanding of brain function. Instead of random noise, we find a fractal order (resembling Lévy flights) in the temporal structure of spontaneous flight maneuvers in tethered *Drosophila* fruit flies. Lévy-like probabilistic behavior patterns are evolutionarily conserved, suggesting a general neural mechanism underlying spontaneous behavior. *Drosophila* can produce these patterns endogenously, without any external cues. The fly's behavior is controlled by brain circuits which operate as a nonlinear system with unstable dynamics far from equilibrium. These findings suggest that both general models of brain function and autonomous agents ought to include biologically relevant nonlinear, endogenous behavior-initiating mechanisms if they strive to realistically simulate biological brains or out-compete other agents.

## Introduction

According to Laplace, randomness is only a measure of our “ignorance of the different causes involved in the production of events.” [1] Probably the most fundamental feature of modern scientific inquiry is the ability to find these causes and predict future events [1], [2]. Reflecting this view, animals are thought to operate according to laws firmly tying behavioral ‘responses’ to environmental variables: “[N]euroscience, over the last 30 years, […] each year brings a greater understanding of the mechanical way with which we perceive, we remember, we speak, we feel.” [3] Once these laws are known, the behavior of any animal at any time can be predicted from the current environmental situation [4]: “We cannot prove […] that human behavior […] is fully determined, but the position becomes more plausible as facts accumulate.” [5] This does not necessarily imply that the same stimulus always elicits the same behavior, but that each behavior is a response to a stimulus: “Indeed, so pervasive is the basic assumption of this model that it is common to refer to any behaviour as a ‘response’ and thus by implication […] assume that there must be an eliciting stimulus.” [6] This basic tenet not only guides basic neurobiological and psychological research but has been the foundation for a great many robotics applications [7]–[9] as well as for speculations on the future societal impact of neuroscience [3], [10], [11]. Basically, the brain is seen an input/output device: “brain function is ultimately best understood in terms of input/output transformations and how they are produced” [12]. Contending that less complex brains would be more amenable to this research, the study of invertebrate and in particular fly behavior developed into a prominent focus of attention [7], [8], [13], [14].

**Figure 1:**
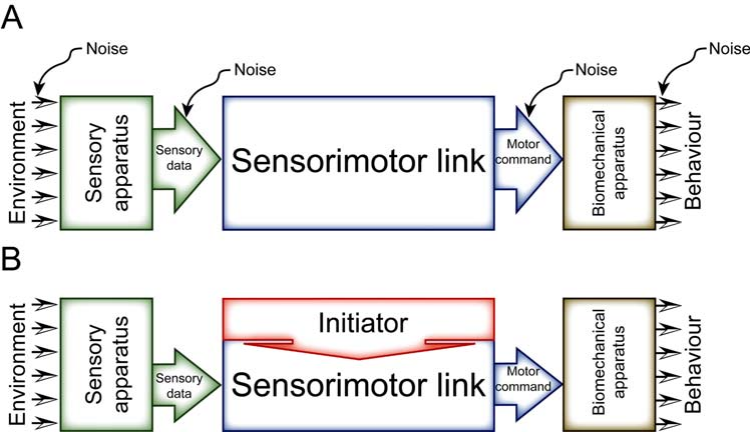
Alternative models conceptualizing the open-loop experiment. A–According to the robot-hypothesis, there is an unambiguous mapping of sensory input to behavioral output. If the behavioral output is not constant in a constant environment, there are a number of possible sources of noise, which would be responsible for the varying output. B–In a competing hypothesis, non-constant output is generated intrinsically by an initiator of behavioral activity. Note that the sources of noise have been omitted in B merely because their contribution may be small, compared to that of the initiator, not because they are thought to be non-existent.

However, even the best-understood behavioral systems display a residual of variability, which has so far prevented exact predictability of individual behavior. There are a number of systems from single neurons and synapses [15], [16] to invertebrate [17], [18] and vertebrate animals including humans [19]–[21], which even generate variable output despite no variations in input at all, leading to difficulties reproducing even tightly controlled experiments [22]. This variability is often classified as random noise, a by-product of a complex brain [23], [24]. Documented sources of noise range from genetic and historical variations [23] to neural noise [24], [25] or stochastic fluctuations in macromolecule number [26]. This noise requires compensatory homeostatic mechanisms to ensure stable neuronal and network function over extended periods of time [27]. Because of the obvious analogy, we term the hypothesis that brains are deterministic input/output systems with added noise the ‘robot-hypothesis’ (Fig. 1a). A less prominent alternative explanation contends that some of the variability is adaptive and irreducible [19], [20], [28]. According to this latter view, individual behavior is fundamentally indeterministic (not fundamentally deterministic but noisy) and precise prediction principally (not only technically) impossible (Fig. 1b). It is critical to emphasize at this point that the processes leading to behavioral indeterminacy may very well be deterministic: indeterministic output of deterministic systems is a well-known phenomenon [29].

**Figure 2:**
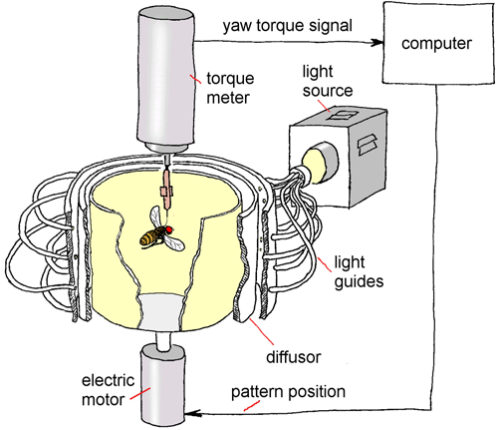
Flight simulator set-up. The fly is flying stationarily in a cylindrical arena homogeneously illuminated from behind. The fly's tendency to perform left or right turns (yaw torque) is measured continuously and fed into the computer. In closed-loop, the computer controls arena rotation (single stripe or uniform texture as patterns on the arena wall). An additional white screen (not shown) covered the arena from above for all groups.

Analyzing the structure of behavioral variability may provide evidence for understanding whether the variability is the result of cumulated errors in an imperfectly wired brain (system noise) or whether the variability is under neural control. In this study, we take advantage of turning behavior in tethered *Drosophila*; this system provides superb control over the perceived environment for a true assessment of the spontaneity of the behavior, while at the same time offering easily quantifiable behavioral dynamics (Fig. 2). Most importantly, we eliminate any potential nonlinear effects which could arise from a closed reafferent feedback loop between the animal's behavior and its environment by opening this loop to study intrinsically generated behavior, without any environmental feedback. Thus, the environment is kept so constant (both between and within experiments), that any remaining minute variation in it must be infinitely smaller than any of the stimuli known to trigger turning behavior [30]. Moreover, the temporal distribution of any such remaining environmental fluctuations can be assumed to be Gaussian. We know of no other intact preparation affording such minute control. We chose the temporal sequence of highly stereotyped flight maneuvers producing short bursts of yaw-torque (‘torque spikes’; corresponding to body-saccades in free flight [31]) for our analysis, because they have been repeatedly both classified as single units of behavior and used for quantitative behavioral analysis. Tethered *Drosophila* produce these spikes in a probabilistic manner not only in response to visual stimulation [14], but also if the stimulus situation is constant [30] (see also Figs. S1 and S2). Freely flying flies do not offer this distinction, as one cannot discern spontaneous body-saccades from elicited body-saccades [32].

## Results

### Spontaneous behavior is not simply random

**Figure 3:**
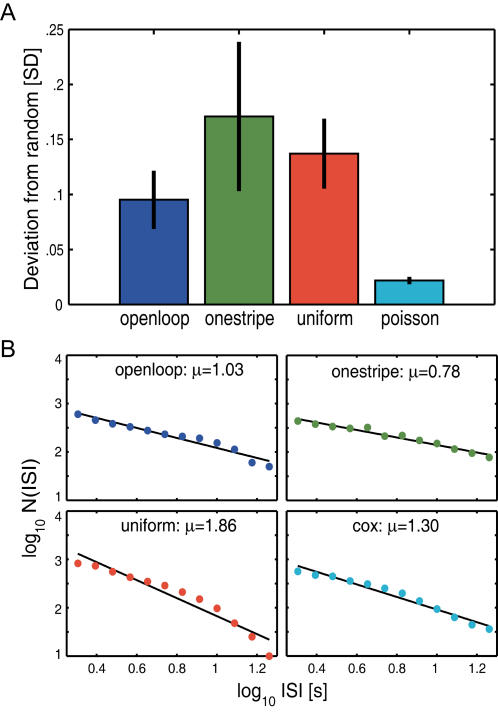
Spontaneous behavior is not simply random. A–GRIP analysis of ISIs. Plotted are the mean standard deviations from the theoretically expected random value for fly ISI series and the random series generated by a Poisson process. The fly deviations are all significantly larger than the values for the computer-generated series. B–Log-log plots of ISIs. The Lévy exponent μ is calculated from the inclination of the linear fit. A Lévy distribution is defined as 1<μ<3. Smaller values indicate a larger proportion of long ISIs. A Cox Process (*cox*) reveals a similar power-law structure as the flies. Error bars are S.E.M.s throughout. See *Methods* for details and statistics.

Naively, if the production of torque spikes in our featureless or uniform environment were due to random noise in the *Drosophila* brain or from any uncontrollable input, the time intervals between spikes (inter-spike interval, ISI) should reflect this stochasticity, much like the hiss of static from a radio between stations. Given a certain mean spike rate, the most straightforward assumption is to expect a stochastic procedure to behave according to a Poisson process [24], [25], [33]. In other words, this situation should represent a natural system for generating random numbers. Therefore, we adapted a recently developed computational method, Geometric Random Inner Products (GRIP) [34], to quantify the randomness of the ISI sequences of three groups of flies. The first group (‘*openloop*’) flew in a completely featureless white panorama (i.e., without any feedback from the uniform environment–open loop). The ISI sequence in these flies must be generated entirely spontaneously. The second group (‘*onestripe*’) flew in an environment that contained a single black stripe as a visual landmark (pattern) in a flight simulator situation that allowed for straight flight in optomotor balance (i.e. the fly could use its yaw torque to control the angular position of the stripe–closed loop). Flies from this group not only received reafferent feedback from the effects their maneuvers had on the angular position of the stripe, but it is also known that such stripes elicit optomotor and fixation responses [35] (see also Fig. S2), providing for an input/output control group. The third group (‘*uniform*’) flew in a uniformly textured environment that was otherwise free of any singularities (i.e., closed loop, the fly could use its yaw torque to control the angular position of the evenly dashed environment). This arrangement also allows for straight flight in optomotor balance but it does not elicit any fixation or directional preferences as the *onestripe* situation. Therefore the *uniform* group constitutes an intermediate case. A significant deviation from ideal randomness in any of these groups would contradict the ‘robot-hypothesis’. GRIP results show that fly behavior deviates from perfect randomness (Fig. 3a). In all our groups, this deviation even exceeds the values from a computer-generated Poisson process (Kruskal-Wallis ANOVA: H(3, N = 52) = 17.2; p<0.0007. In post-hoc tests, all fly values were significantly higher than the *poisson* control values, p<0.03 in all cases). Plotting the number of ISIs as a function of ISI duration reveals an overrepresentation of long ISIs with respect to an exponential distribution (so-called heavy-tailed distributions; see Fig. S3). Thus, the simplest hypothesis that first-order noise underlies variable spike generation in a constant environment has to be rejected.

One may argue that the assumption of a constant spike rate is arbitrary, overly simplistic and that more complex stochastic processes are likely to be at work, even in flies. A well-known example of such stochastic processes is a doubly stochastic Poisson process (or Cox Process) [36], [37]. A Cox process is essentially a Poisson process in which the rate is not constant, but fluctuates randomly. In our example, a fly's spike rate may change in response to uncontrolled, random events in the fly's environment or to random events within the fly. Cox processes can generate heavy-tailed distributions, sometimes also called power-law distributions. Power laws are among the most frequent scaling laws that describe the scale invariance found in many natural phenomena and can be seen as a straight line on a log-log graph of the data. Therefore, we plotted the number of ISIs as a function of ISI duration on a double logarithmic scale. To simulate a Cox process, we used the instantaneous spike rates from the flies in the *openloop* group to drive the rate of a Poisson process (*cox*; see *Methods* for details). A very similar process has previously been used to successfully model the spike trains of neurons such as those in the cat visual cortex [38]. We found inverse power-law distributions both in the timing of fly ISIs and in the *cox* group (Fig. 3b). For the two fly groups without a singularity in the environment (*openloop* and *uniform*) and for the Cox process, the duration of ISIs decayed according to a non-Gaussian Lévy distribution (with the Lévy exponent 1<μ<3). Conspicuously, the Cox process is also Lévy distributed. Do such results provide any leads for investigating the potential mechanisms underlying spontaneous turning behavior?

Lévy flights, a special class of Markov processes, are scale invariant and often associated with power-laws described in many other systems [39]–[41]. A Lévy flight can be conceptualized as a process which first chooses a direction at random and then keeps flying for a distance drawn at random from a Lévy distribution [42]. The Cox process, although not working in this way, still yields a Lévy distribution. It has also been proposed that systems with a large number of nonlinearly coupled subsystems also may exhibit Lévy distributions [43], [44]. Clearly, “the presence of such distributions tells us nothing about the mechanisms that give rise to them” [45]. Notwithstanding, all the more common stochastic processes which can give rise to Lévy distributions imply second-order (or conditional) stochastics. These processes share the property that the conditional probability distribution of the next step depends only on their current state and not on the steps in the past (i.e., no memory). The Cox process is a classic representative of this class of conditional stochastic processes.

### Spontaneous behavior reveals a fractal order

**Figure 4:**
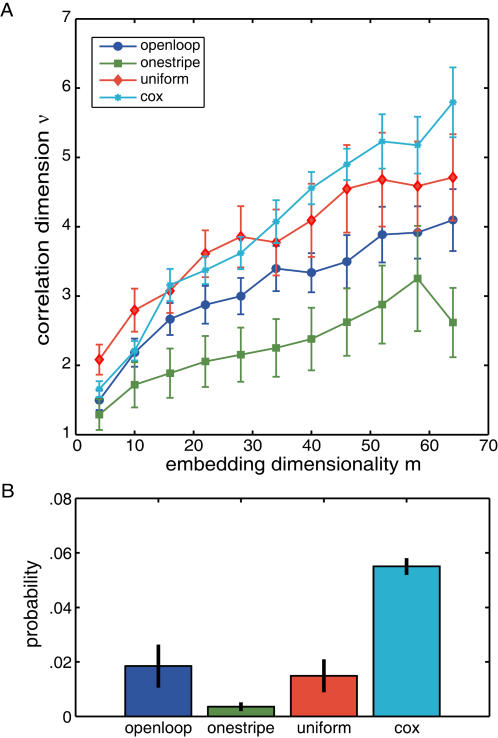
Correlation dimension. A–While the correlation dimension converges on a group-specific value with increasing embedding dimension for fly-generated ISIs (*openloop, onestripe, uniform*), a number sequence generated randomly by a Cox Process (*cox*) diverges. B–Probability to obtain the computed correlation dimensions in A by random shuffling of the original data. While the *cox* group exceeds an alpha value of .05, the three fly groups stay well below that threshold.

A standard method of testing for renewal processes without memory (i.e., Markov, Lévy or Cox processes) is to compare the original sequence to randomly shuffled (“surrogate”) sequences. This surrogate data set maintains the same relative frequency of ISI durations as the original data, but destroys the ordering of the intervals. A significant difference between surrogate data and original data indicates that conditional probabilities are not involved in the generation of the series. For this comparison, we first computed the correlation dimension [46] for the original ISI series which yields a sequence-dependent measure for each fly. The correlation dimension is a measure of the dimensionality of the space occupied by a particular ISI sequence (similar to the less reliable *fractal dimension*). If the correlation dimension converges on a fractional value, the ISI sequence is termed ‘fractal’. This first step of computing individual correlation dimensions already hints at a difference between the stochastic ISI series and the fly series: all four traces appear very similar, but the fly data each converge on a specific dimension while the *cox* series diverges with increasing embedding dimensionality (Fig. 4a). The convergence of the correlation dimensions for fly data suggests a fractal order in the fly ISI series and not in the *cox* series. However, these differences are rather subtle and somewhat subjective. In the decisive second step, we calculated the probability that any randomly shuffled sequence of ISIs could have produced the same outcome. The results show that most likely the recorded sequence of ISIs–and not any random shuffling thereof–is responsible for the computed correlation dimensions, rejecting the hypothesis of second-order stochastics dominating the generation of spontaneous turning behavior in *Drosophila* (Fig. 4b). Similar to sequences of ISIs recorded in the monkey basal ganglia [47], sequences of fly ISIs are not entirely defined by their probability distribution. In contrast, we can not reject the hypothesis that any sequence could generate the computed correlation dimension for the *cox* series, at the .05 criterion. A Kruskal-Wallis ANOVA was significant for the shuffled correlation dimension probabilities: H(3, N = 52) = 24.7; p<0.0001. All fly probabilities were significantly lower than the *cox* probability (p<0.02 in all cases). This outcome rules out renewal processes as the main mechanism generating spontaneous turns in *Drosophila*. Specifically, this excludes Cox processes or other superpositions of random processes, which one could assume if several separate processes in the brain lead to torque spike production or for the superposition of environmentally and endogenously triggered torque spikes.

### Long-range correlations in the behavior imply nonlinearity

**Figure 5:**
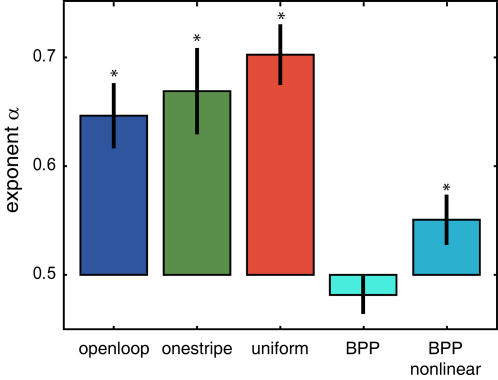
Long-range correlations in fly ISIs. If the slope of the log-log plots of the r.m.s. fluctuation (exponent α, see *Methods*) deviates significantly from ½, long-range correlations exist in the time series. All three fly groups show a significant deviation from 0.5. The deviation of branched Poisson processes (*BPP*), however, depends on the nonlinearity of the filter function used to drive the Poisson processes and is significantly smaller than that of fly ISI series. *-significant difference from 0.5.

However, there are yet more complex composite stochastic models which, like the fly data, can exhibit a fractal structure [15], [48]. These models combine a multitude of stochastic processes by deterministic rules. For instance, the so-called “branched Poisson process” (*BPP*, see Fig. S4a) consists of a cascade of Poisson processes each driving the rate of the next via a filter function [48]. The combined output of all these processes constitutes the output of the entire *BPP*. Such processes can produce ISI series which do show fractal characteristics and their probability of shuffled data to yield the same correlation dimension comes to lie in-between standard stochastics and fly data, such that they cannot easily be distinguished from either of the two (data not shown). The results from surrogate data imply a form of memory in both spontaneous flight behavior and to a certain degree also in *BPP*s that lasts beyond the current time point. Specific ISI durations are determined in part by the timing of other spike(s), and ISI durations fluctuate over time rather than relaxing to a homeostatic steady state. Such a memory can lead to long-range correlations in the data which may be the reason why the shuffled data fail to reproduce the original correlation dimension. A sensitive method to detect these correlations is to calculate the root mean square (r.m.s.) fluctuations in the ISI series (see *Methods*). For uncorrelated time series r.m.s. fluctuations decay according to a power-law with an exponent α of ½. If the exponent deviates from ½, long-range correlations exist in the time series [32], [49]. This computation shows significant deviations from ½ for all the fly series (Fig. 5; t-test against single value: p<0.001 for all three groups). Besides the fly data, we tested two forms of BPP, one with a linear filter function and one with a nonlinear filter. We found that the presence of long-range correlations was dependent on the nonlinearity of the filter function (Fig. 5; t-test against single value: p<0.3 for *BPP* with linear filter and p<0.04 for *BPP* with nonlinear filter). However, the value for the *BPP* with the nonlinear filter function is still significantly smaller than the value for the *openloop* group, to which it was fitted (Mann-Whitney U-Test, p<0.005), ruling out even *BPP*s with nonlinear filters as an appropriate model for spontaneous flight behavior in *Drosophila*.

The dependence of the α-values on the nonlinearity contained in the BPPs entices to hypothesize that what is needed to achieve long-term correlations such as those observed in flies (this study and [32]) and other animals such as albatrosses [49] are not essentially random processes connected by nonlinear mechanisms, but rather essentially nonlinear processes containing random noise. We thus employed a recently developed method which distinguishes essentially stochastic from essentially nonlinear time series.

### Nonlinearity in the behavior implies instability in the brain

**Figure 6:**
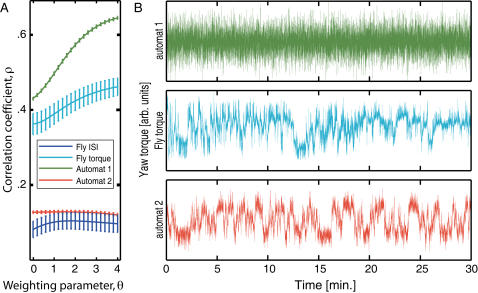
Nonlinearity implies instability. A–S-Map results. Depicted are the averaged results for fly ISIs and raw yaw torque series (for clarity, only *openloop* data are shown here), together with two automat simulations. The fly ISI series shows a slightly improved forecast skill with increasingly nonlinear S-map solutions (increasing Θ). Fly yaw torque series yield both a better overall forecast skill as well as increased nonlinear improvement. The automat simulation can be tuned to produce both linear and nonlinear output. B–Sample raw yaw torque data traces from a real fly and the two versions of the simulated agent depicted in A (automat 1, automat 2). S-Map results for the other two groups are depicted in Fig. S5.

All the previous analyses showed that *Drosophila* turning behavior is at least partially non-random. Information theory tells us that in this case the ISI series contain some sort of information [50]. Forecasting analyses can use this information to predict parts of the sequences. Similar to a weather forecast, forecasting analyses use part of the time series to derive a mathematical model which predicts the remainder of the series. The computed prediction is then compared to the actual series to obtain a correlation coefficient which is a measure for the accuracy of the prediction. Specifically, nonlinear forecasting comprises a set of established methods from nonlinear time series analysis that involve state space reconstruction with lagged coordinate embeddings [51], [52]. These methods take advantage of the loss of information in nonlinear time series to distinguish them from essentially stochastic (high-dimensional, linear) series. In a two-step procedure, we use the Simplex-projection [52] to identify the best embedding dimension and the S-map procedures [53] to assess the nonlinearity of the data (Fig. 6). The method of S-maps relies on fitting a series of models (from linear to nonlinear) where the degree of nonlinearity is controlled by a local weighting parameter Θ. Improved out-of-sample forecast skill with increasingly nonlinear models (larger Θ) indicates that the underlying dynamics were themselves nonlinear [53]. The fly ISI time series show a weak but consistent improved forecast skill with increasing Θ, exhibiting a nonlinear signature (Fig. 6a). However, the overall nonlinear forecast skill is rather low for fly ISI series. To exclude any loss of information introduced by spike detection, we also evaluated the raw yaw torque data series. Analyzing the raw data with the two-step S-Map method also yields increased forecast skill for increasingly nonlinear models, this time with a profoundly larger overall forecast skill (Fig. 6a). This result excludes all essentially stochastic models irrespective of their memory as the basis for fly turning behavior and firmly establishes nonlinearity as the main mechanism.

A popular concept of animal behaviour includes the transition between motivational states. True state shifts are not random features of a time series but instead formally associated with the idea of nonlinearity [54]. Hallmarks of state shifts are e.g. alternative basins of attraction, multiple stable states, hysteresis and fold catastrophe, all of which require the underlying dynamics to be nonlinear in origin [53]. Our analysis suggests that the brain structures generating yaw-torque spikes also operate according to nonlinear rules, similar to the ones discovered in many other natural systems. Nonlinearity is ubiquitous in nervous systems, from single neurons to circuits [29]. A critic may thus argue that the nonlinear signature we find in the fly behavior is merely a reflection of this already well-known property and not indicative of fine-tuned neural control systems. To test this hypothesis, we adapted a virtual agent (i.e., a computer model or *automat*) [55] consisting of three coupled nonlinear generators for comparison with our fly raw data. The agent is intuitively very appealing on a number of levels. First, its structure resembles one which may be expected for fly torque production: one of the generators (the “activator”) activates the other two (“left torque” and “right torque”), which resembles how a motor command from the brain would activate motor patterns in the thoracic ganglion. The two torque generators mutually inhibit each other, preventing the simultaneous activation of right and left turns (Fig. S4b). Second, the original agent's search behavior is similar to a Lévy walk [55]. Third, the *automat* can be tuned so that its open-loop output shows a similar nonlinear signature as fly turning behavior (Fig. 6a, “automat 1”). Fourth, the *automat* can be adjusted such that its output appears to be qualitatively similar to fly open-loop turning behavior (Fig. 6b, “automat 2”). Thus, it seems that indeed the biologically plausible, nonlinear processes in the agent are sufficient to model fly behavior. However, interestingly, if the *automat* is tuned to resemble fly behavior, it does not reveal a nonlinear signature in the S-Map procedure (Fig. 6a, “automat 2”). Indeed, to reveal its nonlinear signature, the *automat* has to be adjusted such that the nonlinear generators operate under unstable conditions, at which point the output fails to resemble fly behavior (Fig. 6b, “automat 1”). This experiment falsifies the initial hypothesis that the nonlinear signature we find in fly behavior is merely a reflection of the well-known nonlinear properties of brains. Nonlinearity is a necessary, but not a sufficient criterion: only if the systems operate under unstable conditions does the output reveal significant nonlinearity (see Fig. S5 for additional S-Map results). The failure of this agent to adequately model fly behavior is an example for the rarely appreciated property of nonlinear systems to produce linear output under equilibrium conditions.

## Discussion

Even small fly brains can control behavior with minute precision. For instance, male house flies closely track the evading flight maneuvers of female flies with only a lag of about 30ms [56]. Input/output models reproduce these chasing flights with high fidelity [56]–[58]. Such input/output systems provide the flies with exquisite control over their turning maneuvers. Nevertheless, bereft of visual input flies produce turning maneuvers, the variability of which would never allow them to stay clear of obstacles, land on food, let alone catch the mate. Where does this variability come from? How does the female fly produce seemingly random turn maneuvers, making it so difficult for the male fly to follow? Obviously, the amount of behavioral variability is in itself variable and must be under the control of the brain. How does the brain do this?

Behavioral variability is a well-known phenomenon. It is so pervasive that the semi-serious Harvard Law of Animal Behavior was coined: “Under carefully controlled experimental circumstances, an animal will behave as it damned well pleases.” It is the source of this variability which is under scrutiny here. The current neuroscientific consensus posits that the source of the variability is noise, rendering the variability random or stochastic. We show here that random noise cannot be the sole source of behavioral variability. In addition to the inevitable noise component, we detected a nonlinear signature suggesting deterministic endogenous processes (i.e., an initiator) involved in generating behavioral variability. It is this combination of chance and necessity that renders individual behavior so notoriously unpredictable. The consequences of this result are profound and may seem contradictory at first: despite being largely deterministic, this initiator falsifies the notion of behavioral determinism. By virtue of its sensitivity to initial conditions, the initiator renders genuine spontaneity (“voluntariness” [30]) a biological trait even in flies.

### Even fly brains are more than just input/output systems

The variability in spontaneous fly turning behavior is not solely due to nonlinearity; rather, the nonlinear processes controlling the behavior also have to operate at just the right parameters to produce instability. Moreover, the number of these nonlinear processes has to be small, as nonlinear signatures disappear with increasing superposition of multiple nonlinear processes [59], [60]. Thus, flies are more than simple input/output machines. Similar to flies, human brains also are notorious for their variability and even devote most of their energy budget to intrinsic processing [21]. Our study supports the hypothesis that the nonlinear processes underlying spontaneous behavior initiation have evolved to generate behavioral indeterminacy: The choice of what behavior to produce in the next moment is rarely determinable exactly, but only probabilistically [17], [19], [20]. Implicitly, game theory, the biological study of choice behavior and neuroeconomics have incorporated this feature on an empirical basis [61]–[65]. If our results from a small fly brain hold also for more complex brains, they suggest that the biological basis of the widespread phenomenon of behavioral indeterminacy can be investigated. For instance, inhibiting neurons forming the ellipsoid body, a neuropil structure in the fly central brain, shifts the temporal structure of *Drosophila* walking behavior from non-Gaussian to Gaussian [41]. It will be interesting to screen for the neurons involved in initiating spontaneous turning behavior as well. Classes of behaviors may be controlled by separate initiators. For instance, human eye saccades show a Gaussian temporal structure [66], whereas communication and travel are clearly non-Gaussian [33], [67], [68]. Also in humans, a “default network” seems to be responsible for spontaneous, stimulus-independent thought [69]. Our data may help explain the notorious difficulty to exactly reproduce behavioral results even when they are under extremely tight experimental control [22]. We hypothesize that the degree to which an animal behaves deterministically is shaped by evolution and thus depends on the ecological niche for which the behavior evolved.

### Optimal searching behavior

What, if any, ecological niche has spontaneous flight behavior in *Drosophila* evolved for? Given the artificial circumstances of our experiments, one would assume that the flies were highly motivated to find an escape. Could the heavy-tailed distribution of turning maneuvers constitute an evolved search behavior? A number of publications have reported Lévy-like search strategies in analyses of a variety of behaviors from plankton to humans [32], [33], [49], [68], [70]. Lévy flights or walks cause the organism to hit a fractal clustered set of points. Surprisingly, flies can in principle produce such behavioral patterns even without any environmental feedback at all (*openloop*, Fig. 3b). One would conclude that internal timing rather than external cues is organizing this behavior. Obviously, environmental feedback can alter the timing of the torque spikes and can thus increase (*uniform*) or decrease (*onestripe*) the distribution characteristics (Fig. 3b). In our setup, the flies can only receive horizontal visual feedback. Nevertheless, the *uniform* group already shows a Lévy exponent very close to the μ≈2 which was observed in freely flying *Drosophila* [32]. Movement patterns with such properties are known to constitute a mathematically optimal search strategy for randomly and sparsely distributed resources [39]. Thus, it appears that all that is required to produce such an optimal search strategy is a default network which spontaneously generates behavior that is already close to optimal, combined with very rudimentary environmental feedback to adjust the default state to the environment at hand. It seems that one component of such a default strategy in *Drosophila* are search spirals, which arise when multiple body-saccades in the same direction are generated with only short ISIs [32] (see also Fig. S2). Conventional experiments with freely moving animals could never have shown this simple relationship. Indeed, in free flight, changes in environmental feedback did not significantly alter the search characteristics [32]. The discovery of near-optimal built-in search strategies enables us now to investigate the brain mechanisms behind optimal foraging in a genetically tractable model organism. Interestingly, these strategies are not random but nevertheless indeterminate.

### New models of brain function

**Figure 7:**
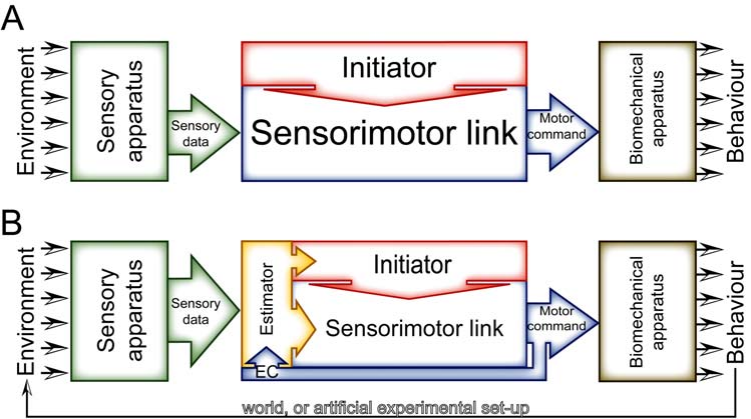
Suggested models for open-and closed-loop experiments. A–Open-loop model as proposed in Fig. 1b (for the *openloop* group). B–Closed-loop model (for the *onestripe* and *uniform* groups). Performance in a situation with a closed reafferent feedback loop is commonly modeled with a state estimator, cross-correlating sensory input with recent motor commands via an efference copy (EC). Such an evaluation is required for efficient behavioral control of incoming sensory data.

Because theoretical work suggests a range of competitive advantages for indeterminate behavior in virtually all animals [19], [61]–[65], [71], the structure of the indeterminacy should be incorporated explicitly into models of general brain function and autonomous agents. What would such future models of brain (or agent) function look like? Nonlinear models displaying probabilistic behavior patterns can in principle be fairly simple [55]. The nonlinear mechanisms need still to be influenced by the environment both in a feed-forward form (the sensorimotor link) [7], [13], [14], [72] and by reafferent feedback control (Fig. 7) [73], [74]. Our data raise the suspicion that future models of the brain may have to implement this or a related component for spontaneous behavior initiation, if they strive to be biologically realistic, out-competing other models/agents. Recently, a new class of agents was introduced, which incorporated some of these ideas [75].

### What is the advantage of nonlinear over random?

But what, if any, difference does it make when behavioral variability–despite being largely unpredictable–is not entirely stochastic, but nonlinear and unstable? The tedious distinction between random noise and unstable nonlinearity is worthwhile, because the former points to extrinsic origins of variability, whereas the latter indicates intrinsic origins. Technical advances frequently lead to a significant increase in signal to noise ratios. Such advances would increase the predictability of a brain where the main source of variability stems from noise. In contrast, noise reductions will only marginally change the predictability of a nonlinear brain whose output is fundamentally indeterministic, despite the deterministic rules that govern it. Given that there is a cost associated with producing indeterminate behavior [61], it is a straightforward inference that these latter rules have evolved specifically to generate varying degrees of behavioral indeterminism [23], as exemplified above in the case of the chasing house flies.

### Brains are simultaneously indeterministic and deterministic for a reason

This insight has implications for our understanding of the general function of brains. The most fundamental brain function is to produce adaptive behavior. Adaptive behavior is the ability to orient toward specific goals in the environment and to control actions flexibly in pursuit of those goals. By and large, the every-day world we live in is Newtonian: predictable and deterministic. If we lose balance, we fall, if we neglect obstacles in our path, we collide with them and if we reach for an object, we can grasp it. Hence, no ambulatory animal could survive without its set of adaptive, hard-wired sensorimotor rules shaped by evolution and tuned by experience. No male house fly would ever catch its mate. At the same time, the world is full of surprises: the unexpected pursuit by a male house fly, the rejection of your manuscript or the next move by your chess opponent (or a predator). In such cases, not even the most complex stimulus-response programs (learned or innate) will help an animal in evading the undesired surprises and obtaining the desired ones. If the evasive actions taken by the female house fly were predictable, males could short cut and catch them with much less effort. It is essential to not leave the generation of behavioral variability to chance (i.e., noise), but to keep it under neural control (i.e., nonlinearity). As such, evolution can fine-tune the balance between sensorimotor mapping and superimposed indeterminacy, defining the required compromise between spontaneous and reactive behavior. The variability of systems under tight constraints will be explained mostly by noise (because the variability under neural control is minimized, such as escape and pursuit responses in flies) [76], whereas noise may play a very small role in generating variability of less constrained behaviors (such as the ones observed here or the evasive actions taken by female house flies) [19], [20], [77]. This notion of brains operating on the critical edge between determinism and chaos has also been used to describe human magnetoencephalographic recordings [78]. Analogous to Heisenberg's uncertainty principle [79], [80], much behavioral variability arises not out of practical constraints, but out of the principles of evolved brain function. In “*What is Life?*” Erwin Schrödinger claimed that fundamental indeterminism would never arise in the living world [81]. Today however, the picture emerges that as much as simple taxis, mate pursuit or course control require deterministic sensorimotor programs [7], [13], [14], [56], [57], [76], more complex interactions require behavioral indeterminism, as evidenced by recent studies in game theory [61], [63], [65], exploration/foraging behavior [71], feeding [82] and pursuit-evasion contests (“Protean Strategy”) [19], [23], [77], [83]. Clearly, deterministic behavior will be exploited [23], [84] and leaves us helpless in unpredictable situations [30], [85]. Brains indeed do throw the dice–but by refuting the notion of stochasticity our results imply that they have exquisite control over when, where and how the dice are thrown [86].

### Spontaneity is the basis for operant behavior

If unpredictability is so important, why is the ‘random number generator’ in the fly brain not perfect? For one, perfect unpredictability might not be required for survival. In addition, variable behavior might serve a second function. Variable, spontaneous behavior is the only way to find out which portions of the incoming sensory stream are under operant control by the animal's behavior. If much of the variation in this stream is due to random noise (i.e., Gaussian), behaving in a non-Gaussian way may aid in the detection of those variations which can be brought under behavioral control. Given these considerations and that our data imply a memory for past events influencing behavior initiation, it is tempting to perceive such mechanisms of spontaneous behavior initiation as the basis for operant behavior, operant conditioning and habit formation [74]. Following this notion, the ecologically so advantageous heavy-tailed searching strategy may be brought about by constantly engaging motor outputs and monitoring their effects in a decision-based queuing process. Such a process prioritizes certain items in a list over others (for instance yaw turns over thrust control, roll or proboscis extension) and has been shown to lead to heavy-tailed behavior patterns [33], [67]. These considerations lend credence to an early, rarely cited cognitive hypothesis on the significance of behavioral variability in vertebrates [28] and suggest that it is actually much more profoundly valid throughout the taxa, with the prospect of studying its biological basis in a genetically tractable model system. Identifying the neural circuitry housing the initiator will be the logical next step in this research.

## Methods

### *Drosophila* at the torque compensator

#### Flies

Flies are kept on standard cornmeal/molasses medium [28] at 25°C and 60% humidity with a 14 hr light/10 hr dark regime. Females aged 24–48 h are briefly immobilized by cold-anaesthesia and glued (Loctite UV glass glue) with head and thorax to a triangle-shaped copper hook (diameter 0.05 mm) the day before the experiment. The animals are then kept individually overnight in small moist chambers containing a few grains of sucrose.

#### Experiments

Fly yaw torque behavior was recorded using a torque compensator [87] with each fly flying stationarily in a vertical drum (arena) as described before [35], [88] for 30 minutes. The *Drosophila* flight simulator is a computer controlled feedback system in which the fly uses its yaw torque to control the rotations of a panorama surrounding it (Fig. 2, Video S1). The core device is the torque meter [35], [89]–[91], which measures a fly's angular momentum around its vertical body axis. The fly, glued to the hook, is attached to the torque meter via a clamp to accomplish stationary flight in the centre of a cylindrical panorama (arena; diameter 58 mm), homogeneously illuminated from behind (Fig. 2). The light source is a 100W, 12V tungsten-iodine bulb.

In the case that the feedback loop between the fly's behavior and its environment is open (i.e., ”open loop”), the arena is empty, stationary and thus supplying a visually constant environment (white light). The fly is stationary, providing for a stable environment in terms of volatiles (odours) and magnetic or electrostatic fields. Any potential auditory stimuli are uncontrolled and bear no correlation to the fly's behavior. An analog to digital converter card (PCL812; Advantech Co.) feeds the yaw torque signal into a computer which stores the trace (sampling frequency 20Hz) for later analysis. 13 flies from this condition form the group “*openloop*”.

In addition to the *openloop* group, we have analyzed data from two control groups. These groups controlled arena positioning with the operant feedback loop between behavior and arena closed. In “closed-loop”, the situation is similar, but differs in that the arena carries either a single stripe (“*onestripe*”) or is uniformly dashed (“*uniform*”). In these cases, a computer controlled electric motor rotates the arena such that its angular velocity is proportional to, but directed against the fly's yaw torque (coupling factor K = −11°/s·10^−10^Nm). This enables the fly to stabilize the panorama and to control its angular orientation. Each of the two groups contains the data from 13 flies. Only 30 minute-long uninterrupted flights in the respective situation are included in the analyses.

### Data series

#### Yaw torque traces

Observing the stored yaw torque traces after the experiment (Fig. S1), it becomes apparent that the behavioral output does not reflect the constancy of the environmental input at all. Instead, the yaw torque signal shows large fluctuations over the entire yaw torque range. In the *openloop* group, there are two sorts of fluctuations: baseline fluctuations and torque spikes. Because of the lack of landmarks, the fly is unable to acquire optomotor balance in order to fly straight, whereas in the two other groups, the pattern(s) on the arena enable straight flight and a constant baseline in optomotor balance (Fig. S1).

#### Torque spikes

In free flight, fruit flies alter flight direction using rapid stereotyped turns termed body saccades [35], [88]. Such saccades can alter flight direction by up to 90° in 50ms with turning velocities exceeding 1000°/s [14], [31], [35], [92]–[96]. The flight path between saccades is comparatively straight [97]. At the torque compensator, these saccades manifest themselves as short bursts of torque (“spikes”). The dynamics of the spikes themselves adjust to tethered flight conditions, but otherwise tethered flight is in many ways very similar to free flight [14], [35], [96]. After low-pass filtering the raw data (6^th^ order Butterworth IIR, passband 6 Hz, stopband 9 Hz) to remove measurement noise, the zero-crossings of the gradient are detected. The time of the zero-crossing is qualified as a spike event if the peak amplitude falls above a given threshold and outside of a given refractory period after the last spike. The time between two successive spikes is stored as inter-spike-interval (ISI). For each detected spike, the direction (left-turning or right-turning) is stored as well (see Fig. S2). A lower cut-off is made at 300 detected spikes to be able to perform meaningful mathematical analysis, discarding all animals failing this criterion. This, as well as all of the following algorithms was implemented in Matlab (The Mathworks Inc., Natick MA, USA).

#### Computer-generated control series

All our algorithms were also applied to computer-generated random ISI series. Standard stochastics predict the outcome of each algorithm for this group of ISI series, which thus provides a valuable control group. For each of the 13 animals from the *openloop* group, a Poisson distribution was fitted to the ISI histogram. Random series with identical length to the *openloop* series were generated by drawing from these distributions, forming the “*poisson*” group.

Releasing the restriction of a constant spike rate, we generated data using a doubly stochastic Poisson process (or *Cox* process) [14], [35], [96]. For each fly from the *openloop* group, we estimated the instantaneous spike rate for each ISI*_i_* by 1/(ISI*_i_*-ISI*_i-1_* ). The distribution of this top-level stochastic process was modeled non-parametrically, i.e. by computing histograms (bin size 10). To generate test data successive values were drawn at random from this top-level distribution. Each randomly drawn value provided the rate for the bottom-level Poisson process generating torque spikes. This process was iterated until the number of ISIs matched the corresponding fly sequence. Thus, both first and second-order statistics were matched in he *openloop* and the *cox* series.

As a model for a more complex composite stochastic process we used a branching Poisson process (BPP) [36], [37]. There are many variants of such composite processes and a number of them are known to generate heavy-tailed probability distributions like the ones we observed in the fly groups. Specifically, we implemented a series cascade of Thomas processes (Fig. S4a): A top-level Poisson process with a constant rate generates a series of events. This series of singular events is filtered through a filter yielding a continuously valued, time varying signal. This is used as the rate for a (non-homogeneous) Poisson process on the next level, which also generates a series of events. This scheme is iterated over all levels. The output of all levels is combined to yield the output of the BPP (hence *branching* PP). For our analyses we generated data using a BPP comprising 10 levels and an initial rate of 0.05. The transfer function of the filter is given by the coefficients [1] in the nominator and [1–0.9] in the denominator, yielding an exponentially decaying impulse response function. Alternatively we used a 5-tap boxcar filter to investigate the effect of (non-)linearity on the properties of the data generated by the BPP.

In addition to ISI time series, we also computer-generated four categories of raw data traces for the nonlinear forecasting procedures:

*I.* A noisy sine function was used for comparison to a linear process. Data of the same length *n* as the yaw torque data were generated by
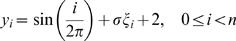
(1)with noise *ξ_i_* drawn from a uniform distribution in the interval [−1, 1]. We set *σ* to 0.2.

*II.* For comparison to a process with known nonlinear properties we used the logistic map: 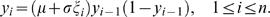
(2)


We chose *μ* = 3.9, *σ* = 0.1, and initialized *y_0_* to a random value in the interval [0, 1].

*III.* We adapted a model designed to simulate spontaneous search behavior as an example for modern autonomous, nonlinear agents. The original model [48] consisted of three coupled nonlinear oscillators and a sensory organ. Two oscillators provided output for left and right turns, respectively. The remaining oscillator provided activating input for the other two oscillators. To model open loop behavior where sensory input is constant, we removed the sensory input from the model (*automat*; Fig. S4b).

The state *s_i_^o^* of oscillator *o* (*o*∈{*R, L, A*} for left, right, and activating) at time point *i* is given by
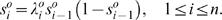
(3)The initial state *s^o^*_0_ of an oscillator is randomly chosen in the interval [0,1]. We re-set *s_i_^o^* to 10^−6^ whenever it falls below this value.

The parameters *λ^o^* evolve according to
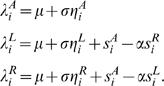
(4)


Here, *η^o^* is Gaussian noise in the interval [−1, 1]. The model parameter *μ* controls the behavior of the logistic maps. The term *ση_i_^o^* acts as a perturbation on *μ*. The parameter *α* controls the strength of the inhibition between the left and right turn oscillators. The simulated torque signal *y* is computed by
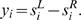
(5)


The model parameters *μ*, *σ*, and *α* were adjusted in the following ways to generate a number of different *automat* simulations. At first, the parameters were chosen according to the original publication (*μ = 1.1*, *σ = 1.1*, and *α* = 1; *automat* in Fig. S5). From there, parameters were explored and adjusted manually until the output appeared to be indistinguishable from fly yaw torque data (*μ* = 1.1, *σ* = 0.75, and *α* = 1.15; *automat 2* in Fig. 6). For this simulation, the previous time-step was also added to the current state (i.e., *s^A^_i_*_−1_+*s_i_^A^*), simulating a one-step memory. Next, *μ* was increased and *σ* decreased to bring the agent beyond the point of stability (*μ* = 3.4, *σ* = 0.3, and *α* = 3.4, *automat 1* in Fig. 6).

### Mathematical analyses

In a stepwise fashion we tested increasingly more sophisticated models, eliminating the less complex models at each step.

#### Geometric Random Inner Products (GRIP)

The GRIP formalism has been developed to quantify the performance of random number generators [55]. It is based on the observation that the average inner product of randomly distributed vectors in n-dimensional geometric objects (like hyper-spheres or hyper-cubes) converges to object specific constants. The deviation from this constant can be used as a measure for the randomness of a sequence. One application was studying the randomness of the digits of π [34].

Here we apply GRIP to quantify the randomness of ISI sequences. In a first step, the ISI sequence (l_1_, l_2_, … l_n_) is embedded in an *d*-dimensional space such that 
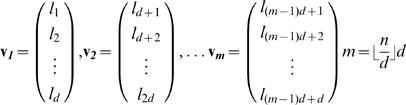
(6)are vectors which are presumed to be random. For three consecutive vectors **v**_i_, **v**_i+1_, **v**_i+2_ the differences **v**_12_ = **v**_i+1_−**v**_i_ and **v**_23_ = **v**_i+2_−**v**_i+1_ are computed. The average inner product of these vectors has been shown to converge to a geometric constant *c_d_*, i.e.
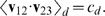
(7)


For an exponential probability density function *p*(*l*) = *e^−al^* of ISIs of length *l*, this constant is
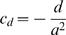
(8)(Tu, S.J.; personal communication). We set the embedding dimension *d* = 3. Exponential functions were fitted to the ISI histograms, and the geometric constants *c_d_* were determined for each fly. To compare the randomness between groups, we computed the absolute differences between the left and right side of eq. (7) in terms of standard deviations of the left side. The results were averaged for each group.

#### Exponential distributions

We compared ISI series to exponential distributions by first fitting an exponential distribution to the ISI series and then plotting the ISI series on a semi-logarithmic scale with the fitted exponential as a straight line. Wherever the ISI series deviates from the straight line, it deviates from an exponential distribution with the same rate.

#### Lévy exponent

If the distribution of ISIs of duration *l* can be characterized by a probability density function
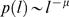
(9)with 1<*μ*≤3, the distribution is called a Lévy distribution. In contrast to Gaussian or Poisson distributions of step lengths, in Lévy motion small steps are more often interspersed with longer steps, causing the variance of the distribution to diverge. Additionally, Lévy distributions are self-similar at all scales or, in other words, the step lengths have no characteristic scale [98]. Lévy distributions are commonly found in animal behavioral patterns [99]. For foraging behavior it can be shown that *μ*≈2 results in an optimal coverage of an area with randomly located target sites if the global site concentration is low [39]. We determined Lévy exponents by fitting straight lines to log-log plots of ISI histograms:
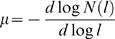
(10)


Here, *N(l)* is the number of ISIs in the bin representing duration *l*. All single fly series within one group were concatenated and *μ* computed as a single value for each group.

#### Correlation dimension

To evaluate the possibility that the apparently random ISI sequences are produced by a nonlinear system causing chaotic dynamics we estimated the fractal dimensions of the underlying attractor of the sequences. Specifically, we computed the limit of the correlation dimension ν for an increasing dimensionality *d* of the embedding space [100],
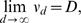
(11)where *D* is the fractal dimension of the chaotic attractor. The correlation dimension is given by:
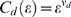
(12)


*C_d_* is the correlation integral and measures how frequently the system state returns into a vicinity of size *ε*,
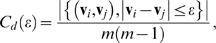
(13)where vectors **v**_1…m_ are the embedded ISI sequence of dimensionality *d*. Similarly, we computed the limit of the information dimension 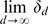 defined as[101]:
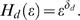
(14)


*H_d_* is the entropy of the system in phase space and can be written as
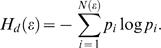
(15)


Here, *p_i_* is the probability that the system is in state *i* represented by cubes of size *ε* in the state space. Numerically, correlation and information dimension were determined by fitting lines into log-log plots of the correlation integral and the entropy, respectively.

For random sequences the correlation dimension diverges. Since we observe convergence for our ISI sequences, we use this as another indicator that they are not trivially random. In order to exclude more complex stochastic processes, we compared the correlation dimension for each dataset with the values obtained from surrogate data. Surrogate datasets were created by randomly shuffling the ISIs of the measured sequence. This retains the first order statistics but destroys any dynamic information depending on the history of the system. If the correlation dimension of the measured sequence and the surrogate data differ significantly, we can conclude that the sequence contains dynamic information. To evaluate the difference we computed a normalized histogram of correlation dimensions of N = 1000 surrogate datasets. In this histogram, the value at the position of the correlation dimension of the measured sequence corresponds to the probability to obtain this value by a random sequence with the same first order statistics. These probabilities were averaged across individuals for each group.

#### Root-mean-square fluctuation of displacement

To detect long-range correlations in our ISI series, we applied a method based on the root mean square (r.m.s.) fluctuation of displacement [102]. If (l_1_, l_2_, … l_n_) is a sequence of ISIs, the net displacement *y(t)* is defined as the running sum 
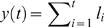
. The fluctuation of displacement is defined as Δ*y*(*t*)≡*y*(*t*_0_ *+t*)−*y*(*t*_0_), and the statistical measure characterizing the series is the root of the mean squares
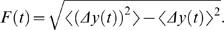
(16)


The angular brackets denote expectation value over all possible values *t_0_*. The r.m.s. fluctuation obeys a power law, i.e.
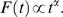
(17)


Uncorrelated time series yield α = ½, as do Markov processes for sufficiently large *t*. Processes with long-range correlations yield α≠½. We plotted *F(t)* for each ISI series on a double logarithmic scale, fitted straight lines and calculated the regression slope α to obtain one value for each series which was then averaged for each group.

#### Simplex projection

Simplex projection [49] is a nonlinear method for making short-term forecasts of time series. The quality of the forecast is measured by computing the correlation coefficient between the forecast and the original series. Depending on the nature of the data, the evolution of the correlation coefficient shows different developments for increasing forecasting intervals. For a linear, but noisy process the correlation coefficient decreases only slowly with increasing prediction intervals. In contrast, a chaotic process is characterized by a fast decay of prediction accuracy. One of the great advantages of this method is that it can be applied to short series, such as our data. Raw yaw torque data were detrended by taking the first difference of the series. ISI series were not detrended.

The method starts by embedding the ISI or data sequence in a *d*-dimensional space. Unlike the embedding used for GRIP and correlation dimension, where each ISI is used in only one vector, here each ISI appears in *d* vectors. Specifically, the embedding is now 
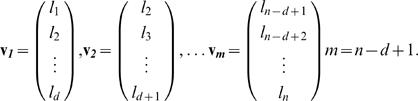
(18)


The resulting set of points in d-dimensional space is split in two halves, the library set **L** and the prediction set **P**. We consider each vector **p** *^t^*∈**P** as composed of a consecutive sequence of *d* observed ISI points. From this sequence a prediction about the following ISI durations (*T_p_* = 1, 2, …) is to be generated. From eq. (18) can be seen, that if **p** *^t^* = **p** *_i_* is the *i*-th vector in the prediction set, the observed ISI *T_p_* steps ahead is 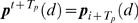, e.g. the prediction for the sequence in **v**_1_ one step ahead is **v**_2_(*d*).

To generate a prediction for a vector **p** *^t^* from the prediction set, its *d*+1 nearest neighbors **l** *^t^*_1_…**l** *^t^_d_*_+1_∈**L** are selected. Associated with each neighbor is a weight
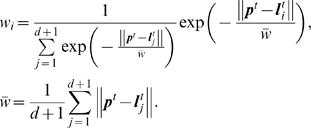



A prediction 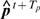 for **p** *^t^* after *T_p_* steps is then given by the weighted superposition of the evolution of the neighbors after *T_p_* time steps, i.e.
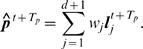



Returning back from the embedding space to the temporal domain of the sequence, we consider the predicted ISI,
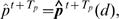
(21)i.e. the last component of vector 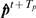, and compare it with the observed ISI *T_p_* steps ahead, which is given by
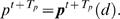
(22)


The coefficient of correlation between the sequence of predicted ISIs and the true values is then used as a measure for the prediction accuracy.

#### S-map procedure

The S-map procedure (sequentially locally weighted global linear map [52]) is in many respects similar to the simplex projection. Here, instead of looking at the evolution of only the nearest neighbors to generate a prediction, *all* vectors in the library set are used. A single linearity parameter *θ* controls if the influence of the library vectors is linear (*θ* = 0) or nonlinearly weighted by their respective distance to the vector used for the prediction. To apply the denotation used for the simplex projection, the prediction 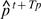 from a vector **p** *^t^*∈**P** is now given by
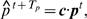
(23)where **c** is a weight vector that is newly computed for every prediction **p** *^t^*. It is the solution of 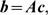
(24)where the rows of matrix **A** contain the library vectors **l** and vector **b** the corresponding, observed ISI duration *T_p_* time steps after the sequence contained in **l**. Formally, **A** and **b** are given by 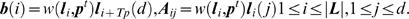
(25)


As can be seen, the number of rows in **A** (and the length of **b**) is equal to the size of the library set |**L**|, which in most cases will be larger than the embedding dimension *d*. Therefore, eq. (24) will be over-determined and singular value decomposition (SVD) is used to obtain an optimal solution.

Function *w* is used to weight the library vectors by their distance to the prediction vector:
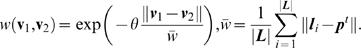
(26)


For *θ* = 0, a linear map is obtained. Increasing *θ* puts more and more emphasis on library vectors close to the prediction vector.

As for the simplex projection, the accuracy of predictions is evaluated by the correlation coefficient between the predicted and the observed series.

### Statistical evaluation

To test for significant differences between several groups, we first used a Kruskal-Wallis ANOVA to test the hypothesis that all groups were drawn from the same population. If this hypothesis was rejected, 2-tailed post-hoc tests provided information as to the source of the differences. These tests were conducted for GRIP values (Fig. 3) and the probabilities to obtain the original correlation dimension with shuffled data (Fig. 4). T-tests against single values were used to test individual groups against an expected value and Mann-Whitney U-Tests for pairwise comparisons (Fig. 5).

## Supporting Information

Figure S1 Example yaw torque traces. Left column-total traces. Right column-magnified section from minutes 5-10 of the total traces. Red lines delineate enlarged sections. Upper row is from an animal flying in open loop in a featureless, white panorama (openloop). The middle row is from an animal flying in closed loop in a panorama with a single black stripe (onestripe). The lower row is from an animal flying in closed loop in a uniformly dashed arena (uniform).
(0.68 MB TIF)

Figure S2 Descriptive statistics of spiking behavior. A-The probability to perform consecutive spikes in the same direction. Random spike directions show equal probability for left and right turns, while fly data are dependent on the environmental situation of the fly. Flies fixate a single stripe and hence produce alternating spikes to keep the stripe in front of them. The onestripe group therefore is more similar to the poisson group than the other fly groups. Flies in uniform environments show persistent turning direction over several consecutive spikes. These spike trains in the same direction can be interpreted as search spirals. B-Total number of spikes. Openloop and poisson show the same values, because poisson was generated by drawing series with the same length as those in openloop. The onsestripe group shows fewer spikes, because of the long intervals flying straight towards the stripe.
(0.89 MB TIF)

Figure S3 Log-linear plots of fly and Poisson data. Corroborating the results from our GRIP analysis, exponential distributions (straight black lines) cannot be fitted to fly ISI series, whereas the poisson series shows the expected exponential distribution. Fly ISI series all show an excess of long intervals, suggesting a heavy-tailed distribution. See Methods for details.
(0.38 MB TIF)

Figure S4 Schematic diagrams of complex stochastic and simple nonlinear models. A-The branching Poisson process (BPP) as an example for complex stochastic models. The BPP consists of cascading units of filter functions and Poisson processes. Each unit's filter function receives the events from the Poisson process upstream and drives the rate of the Poisson process associated with it. The (unfiltered) output of all Poisson processes is combined to yield the total output of the model. B-The nonlinear automat is an example how simple nonlinear processes can generate complex behavior. The activator sends excitatory input to both turn generators. The turn oscillators inhibit each other. The output is the difference signal between the left and right turn oscillator. Each oscillator is described by a logistic map, and the coupling modulates the individual parameters of each map. See Methods for details.
(1.44 MB TIF)

Figure S5 S-Map analysis of all fly data and additional control series. A-S-Map analysis of ISI series. Depicted are the averaged results for the three fly groups. Interestingly, the fly group with a singularity in the environment (onestripe) can be clearly distinguished from the two groups with uniform environment (openloop and uniform). Note that the closed-loop groups (onestripe and uniform) also exhibit the nonlinear signature, excluding the possibility that the variability is an artefact of the constant stimulus situation in the openloop group. B-S-Map analysis of raw data series. At high parameter values, the logistic map shows the typical increase in forecast skill with increasingly nonlinear models, while the noisy sine function does not show any such improvement. The nonlinear agent (automat) with the originally published parameters behaves almost randomly, despite the nonlinear mechanisms generating the output. The fly data come to lie in-between the extreme control data, showing both an increase in forecast skill with increasingly nonlinear models and moderate overall correlation coefficients.
(0.42 MB TIF)

Video S1 Tethered Drosophila. Tethered flying Drosophila can beat its wings, move its abdomen, legs and proboscis, but cannot rotate or otherwise move.
(1.94 MB AVI)
