# Supplementary figures and images for: Order in Spontaneous Behavior
Source: PLoS One. 2007 May 16;2(5):e443. doi: 10.1371/journal.pone.0000443 (PMC1865389; doi:10.1371/journal.pone.0000443)

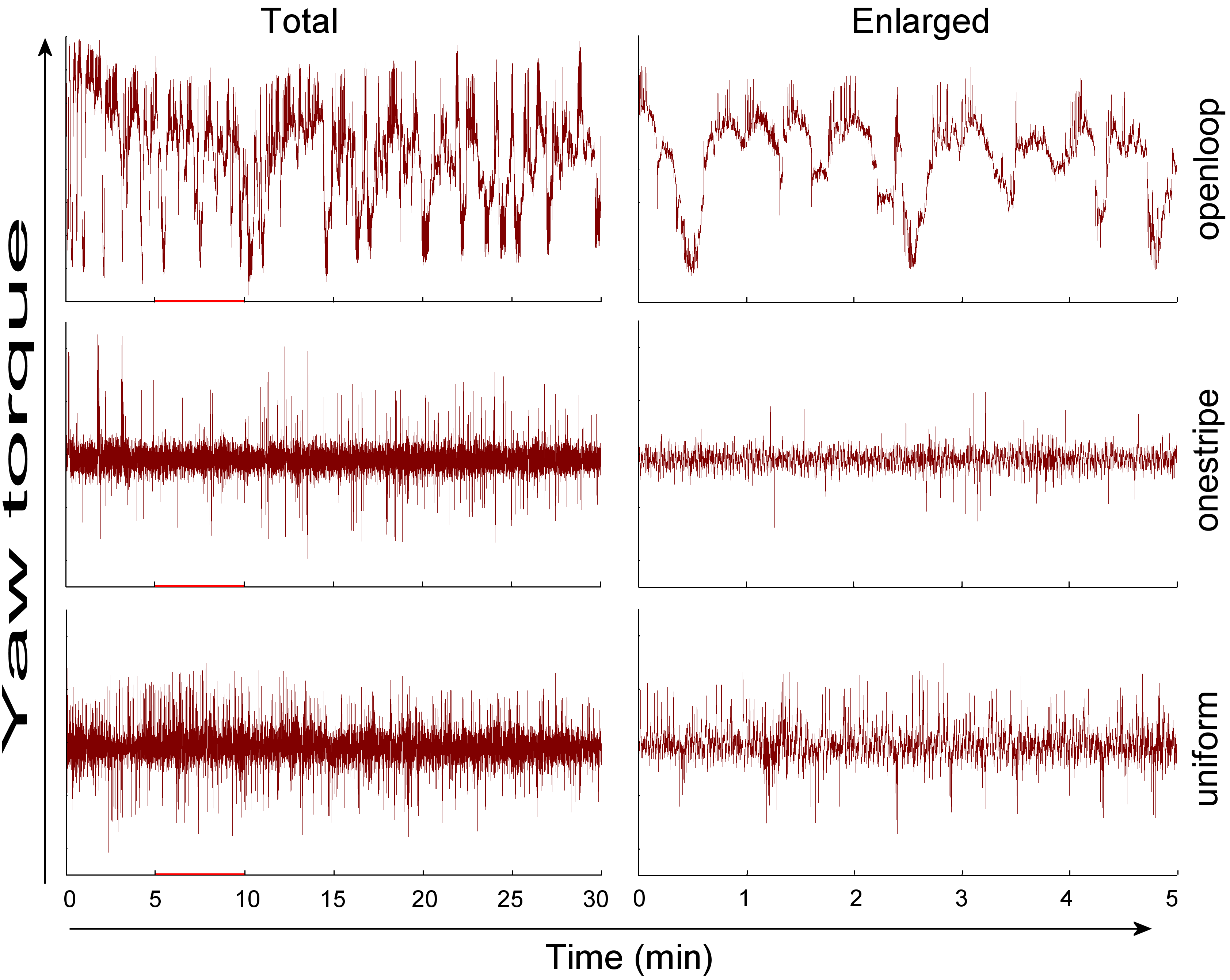

Supplement: Figure S1 — Example yaw torque traces. Left column-total traces. Right column-magnified section from minutes 5-10 of the total traces. Red lines delineate enlarged sections. Upper row is from an animal flying in open loop in a featureless, white panorama (openloop). The middle row is from an animal flying in closed loop in a panorama with a single black stripe (onestripe). The lower row is from an animal flying in closed loop in a uniformly dashed arena (uniform). (0.68 MB TIF) [file pone.0000443.s001.tif]

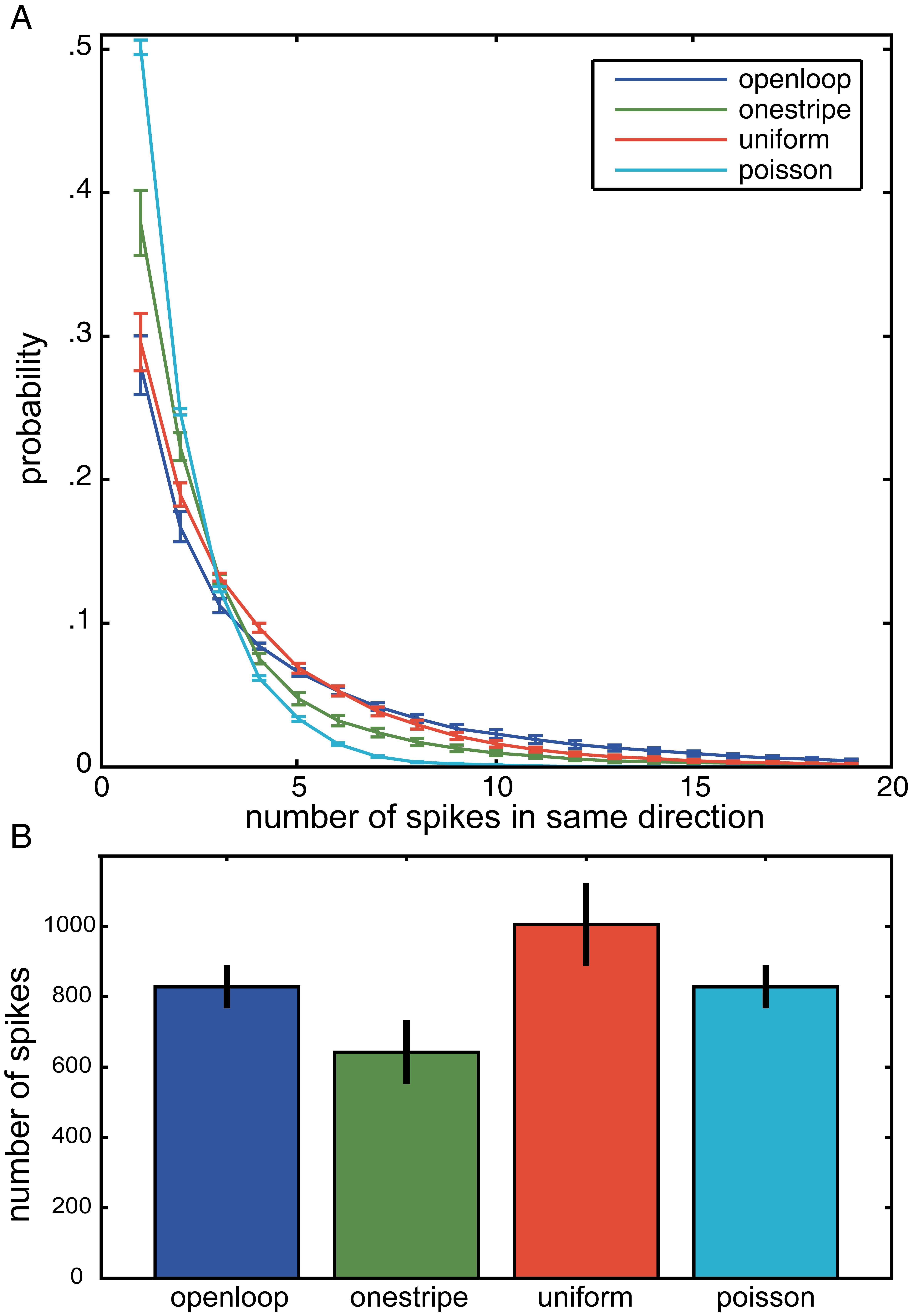

Supplement: Figure S2 — Descriptive statistics of spiking behavior. A-The probability to perform consecutive spikes in the same direction. Random spike directions show equal probability for left and right turns, while fly data are dependent on the environmental situation of the fly. Flies fixate a single stripe and hence produce alternating spikes to keep the stripe in front of them. The onestripe group therefore is more similar to the poisson group than the other fly groups. Flies in uniform environments show persistent turning direction over several consecutive spikes. These spike trains in the same direction can be interpreted as search spirals. B-Total number of spikes. Openloop and poisson show the same values, because poisson was generated by drawing series with the same length as those in openloop. The onsestripe group shows fewer spikes, because of the long intervals flying straight towards the stripe. (0.89 MB TIF) [file pone.0000443.s002.tif]

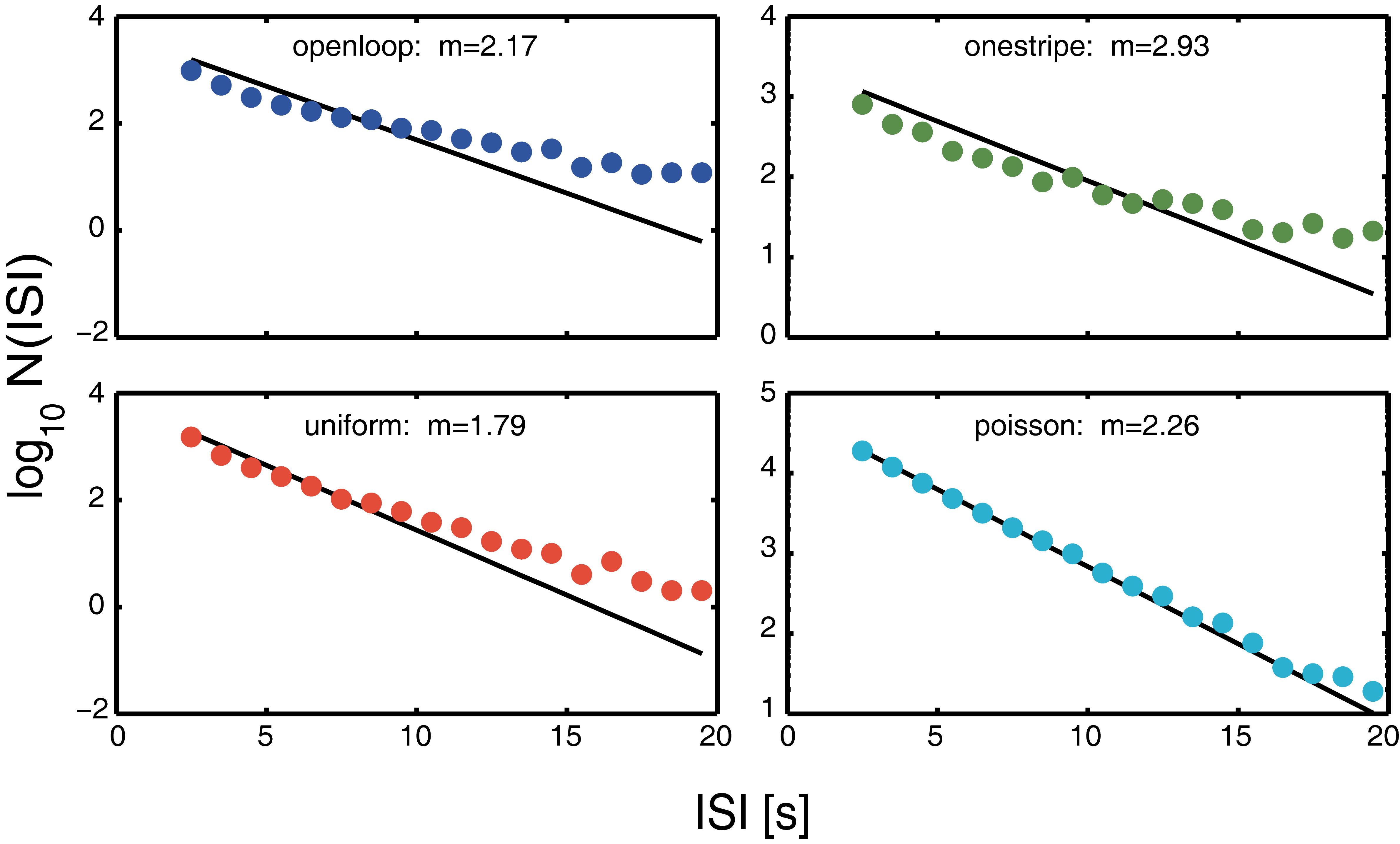

Supplement: Figure S3 — Log-linear plots of fly and Poisson data. Corroborating the results from our GRIP analysis, exponential distributions (straight black lines) cannot be fitted to fly ISI series, whereas the poisson series shows the expected exponential distribution. Fly ISI series all show an excess of long intervals, suggesting a heavy-tailed distribution. See Methods for details. (0.38 MB TIF) [file pone.0000443.s003.tif]

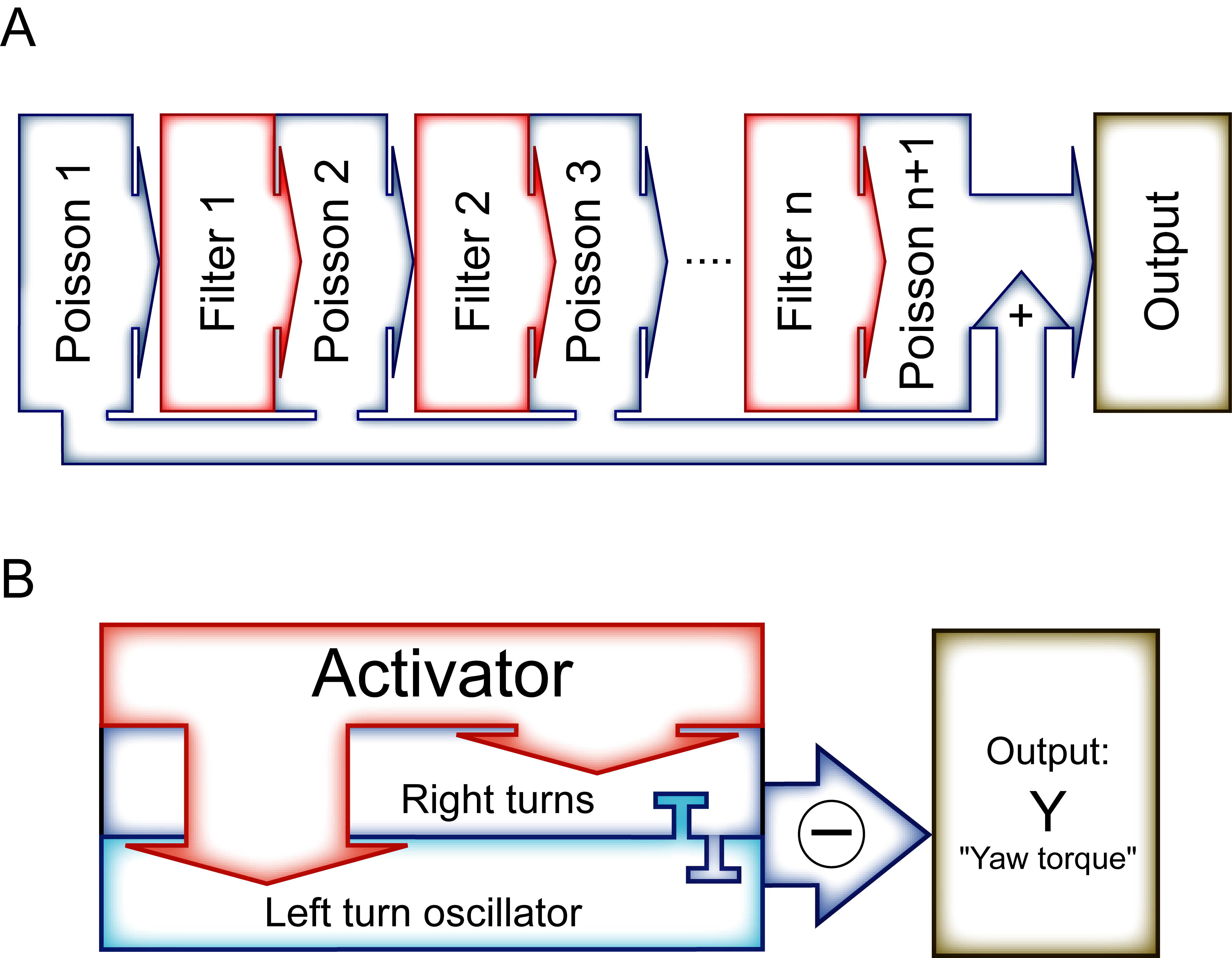

Supplement: Figure S4 — Schematic diagrams of complex stochastic and simple nonlinear models. A-The branching Poisson process (BPP) as an example for complex stochastic models. The BPP consists of cascading units of filter functions and Poisson processes. Each unit's filter function receives the events from the Poisson process upstream and drives the rate of the Poisson process associated with it. The (unfiltered) output of all Poisson processes is combined to yield the total output of the model. B-The nonlinear automat is an example how simple nonlinear processes can generate complex behavior. The activator sends excitatory input to both turn generators. The turn oscillators inhibit each other. The output is the difference signal between the left and right turn oscillator. Each oscillator is described by a logistic map, and the coupling modulates the individual parameters of each map. See Methods for details. (1.44 MB TIF) [file pone.0000443.s004.tif]

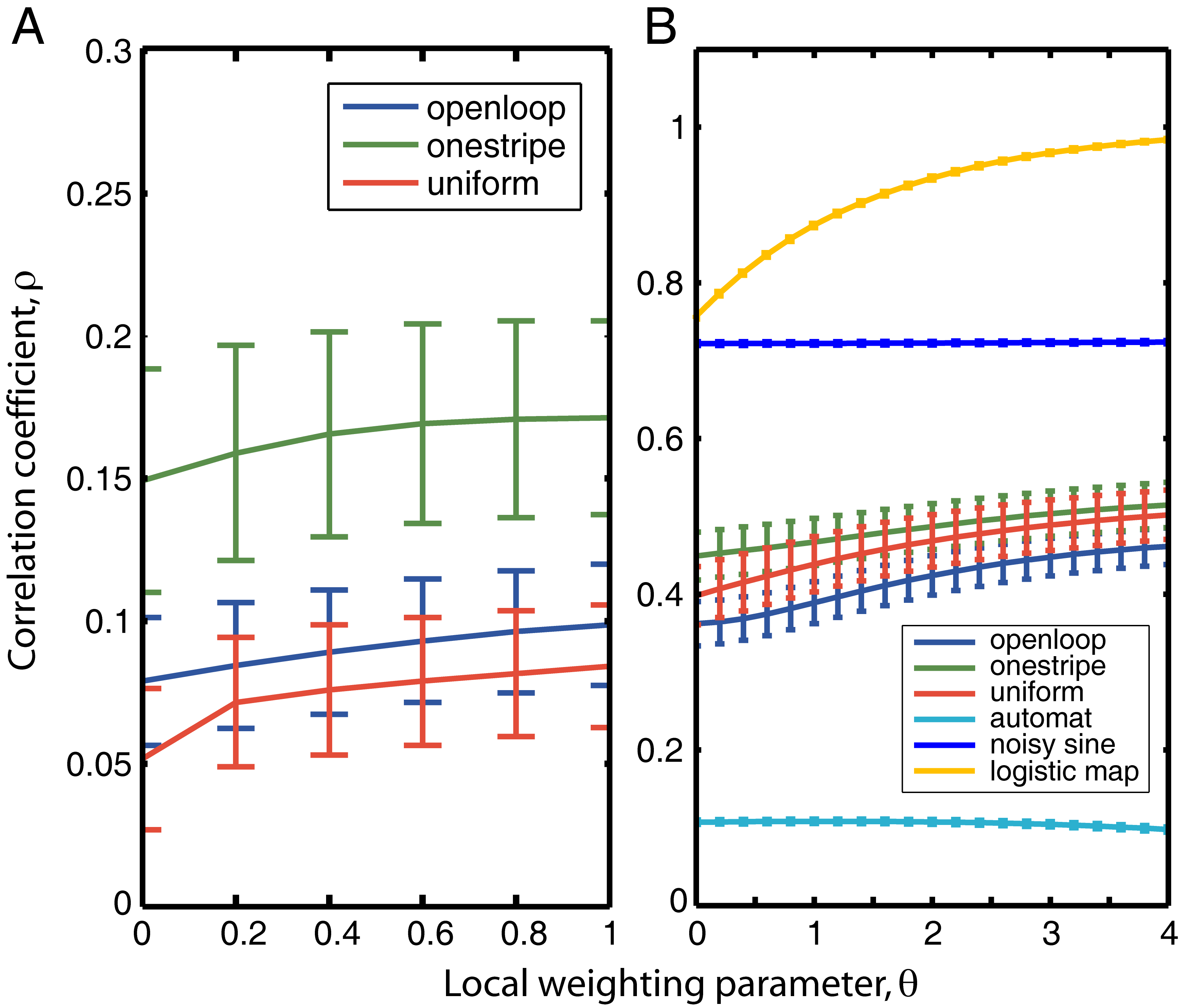

Supplement: Figure S5 — S-Map analysis of all fly data and additional control series. A-S-Map analysis of ISI series. Depicted are the averaged results for the three fly groups. Interestingly, the fly group with a singularity in the environment (onestripe) can be clearly distinguished from the two groups with uniform environment (openloop and uniform). Note that the closed-loop groups (onestripe and uniform) also exhibit the nonlinear signature, excluding the possibility that the variability is an artefact of the constant stimulus situation in the openloop group. B-S-Map analysis of raw data series. At high parameter values, the logistic map shows the typical increase in forecast skill with increasingly nonlinear models, while the noisy sine function does not show any such improvement. The nonlinear agent (automat) with the originally published parameters behaves almost randomly, despite the nonlinear mechanisms generating the output. The fly data come to lie in-between the extreme control data, showing both an increase in forecast skill with increasingly nonlinear models and moderate overall correlation coefficients. (0.42 MB TIF) [file pone.0000443.s005.tif]
